# Modern Stereotactic Radiotherapy for Brain Metastases from Lung Cancer: Current Trends and Future Perspectives Based on Integrated Translational Approaches

**DOI:** 10.3390/cancers15184622

**Published:** 2023-09-18

**Authors:** Mario Levis, Alessio Gastino, Greta De Giorgi, Cristina Mantovani, Paolo Bironzo, Luca Mangherini, Alessia Andrea Ricci, Umberto Ricardi, Paola Cassoni, Luca Bertero

**Affiliations:** 1 Radiation Oncology Unit, Department of Oncology, University of Turin, 10126 Turin, Italy; mario.levis@unito.it (M.L.); alessio.gastino@gmail.com (A.G.); gre21.92@gmail.com (G.D.G.); cristina.mantovani@ymail.com (C.M.); umberto.ricardi@unito.it (U.R.); 2 Oncology Unit, Department of Oncology, San Luigi Gonzaga Hospital, University of Turin, 10043 Orbassano, Italy; paolo.bironzo@unito.it; 3 Pathology Unit, Department of Medical Sciences, University of Turin, 10126 Turin, Italy; luca.mangherini@unito.it (L.M.); alessiaandrea.ricci@unito.it (A.A.R.); paola.cassoni@unito.it (P.C.)

**Keywords:** NSCLC, brain metastasis, oligometastatic disease, radiotherapy, radiosurgery, SRS, molecular profiling, micro-environment, TKI, immune checkpoint inhibitors

## Abstract

**Simple Summary:**

In the current era of precision medicine, the management of patients with brain metastases (BMs) is rapidly evolving. The technical evolution of radiotherapy, now able to offer focal ablative treatments, in combination with new systemic target therapies, is changing the therapeutic landscape in this challenging clinical setting. Moreover, the emerging role of the tumor microenvironment in influencing the response to standard therapies is revealing new potential prognostic and predictive biomarkers for patients with BMs. In this review, we offer an overview of the current trends in the treatment of BMs from lung cancer, with a secondary focus on future perspectives based on integrated translational approaches.

**Abstract:**

Brain metastases (BMs) represent the most frequent metastatic event in the course of lung cancer patients, occurring in approximately 50% of patients with non-small-cell lung cancer (NSCLC) and in up to 70% in patients with small-cell lung cancer (SCLC). Thus far, many advances have been made in the diagnostic and therapeutic procedures, allowing improvements in the prognosis of these patients. The modern approach relies on the integration of several factors, such as accurate histological and molecular profiling, comprehensive assessment of clinical parameters and precise definition of the extent of intracranial and extracranial disease involvement. The combination of these factors is pivotal to guide the multidisciplinary discussion and to offer the most appropriate treatment to these patients based on a personalized approach. Focal radiotherapy (RT), in all its modalities (radiosurgery (SRS), fractionated stereotactic radiotherapy (SRT), adjuvant stereotactic radiotherapy (aSRT)), is the cornerstone of BM management, either alone or in combination with surgery and systemic therapies. We review the modern therapeutic strategies available to treat lung cancer patients with brain involvement. This includes an accurate review of the different technical solutions which can be exploited to provide a “state-of-art” focal RT and also a detailed description of the systemic agents available as effective alternatives to SRS/SRT when a targetable molecular driver is present. In addition to the validated treatment options, we also discuss the future perspective for focal RT, based on emerging clinical reports (e.g., SRS for patients with many BMs from NSCLC or SRS for BMs from SCLC), together with a presentation of innovative and promising findings in translational research and the combination of novel targeted agents with SRS/SRT.

## 1. Introduction

Brain metastases (BMs) represent a central issue in the management of lung cancer, occurring in up to 50% of non-small-cell lung cancer (NSCLC) and over 70% of small-cell lung cancer (SCLC) patients during the course of their disease [1,2]. The incidence and prevalence of BMs are globally increasing due to the advances in diagnostic and therapeutic procedures that chronicize the natural history of lung cancer [3].

Considering the heterogeneity of presentations in this setting, an accurate prognostic definition is crucial to offer personalized therapies. The evolution of prognostic indices reflects the relevant changes that have occurred in the clinical practice of treating BMs. The original recursive partitioning analysis (RPA) was defined in the era of the “one size fits all approach”, when whole brain radiation therapy (WBRT) was considered the standard treatment for nearly all patients. At that time, only clinical factors such as age, Karnofsky performance status (KPS), status of the primary tumor and presence of extracranial metastases were included in the model [4]. The subsequent graded prognostic assessment (GPA) introduced the number of brain lesions, which underscored the importance of local treatments by surgery and focal radiation therapy (RT) in patients with a limited number of metastases [5]. Finally, the modern lung-cancer-specific GPAs (DS-GPA and Lung-molGPA) stratified the prognosis based on primary histology and molecular subtypes, highlighting the growing role of biomolecular diagnostic and systemic therapy (ST) in this scenario [6,7,8].

In the era of precision medicine, stereotactic radiosurgery (SRS) has gradually replaced the historical WBRT approach as a safer and more effective technique, and today represents a cornerstone of BM management [9].

Our review aims to offer an overview of the modern SRS strategies available to treat BMs from lung cancer, either alone or in combination with surgery and ST. In addition, we also describe in detail the molecular profile of lung cancer BMs and the emerging understanding of the role of the tumor microenvironment in influencing the response to standard therapies, revealing new potential prognostic and predictive biomarkers.

## 2. Stereotactic Radiosurgery (SRS)

SRS is a highly conformal technique, able to deliver a high dose of radiation in a single fraction to a well-defined target. This ablative and focal strategy mimics the effect of surgical resection through a less invasive approach, representing the ideal solution for small-sized BMs and a valid alternative when surgery is not feasible (generally for unresectable lesions critically located in deep or eloquent brain areas [10,11,12] or patients inoperable due to comorbidities).

### 2.1. Technical Solutions, Doses and Fractionations

**Figure 1:**
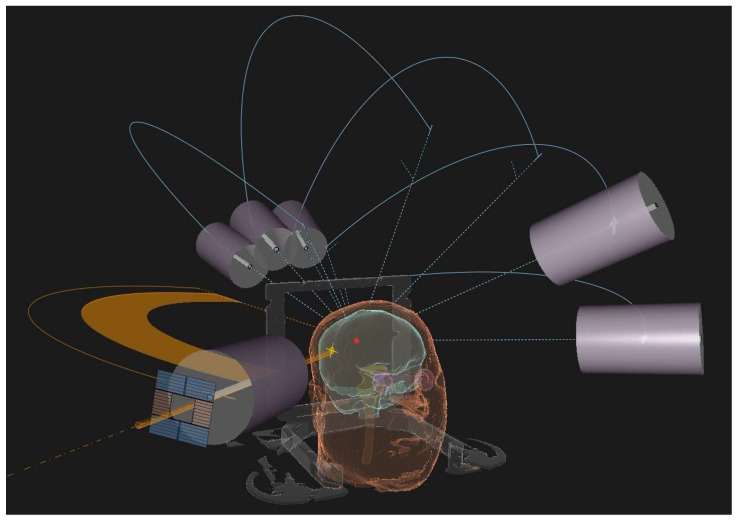
3D treatment planning reconstruction of a LINAC-based SRS treatment using conical-shaped collimators.

Linear accelerators (LINACs), the Gamma Knife and Cyberknife are the platforms usually adopted for the radiosurgical treatment of BMs (Figure 1 shows a LINAC-based SRS approach).

Over the decades, many technological advances have guaranteed a less invasive and more accurate delivery of SRS, and no clear advantages emerged in comparisons among these technical solutions in terms of efficacy or toxicity [13,14].

Different doses and fractionations have been evaluated and are adopted in clinical practice according to a risk-adaptive approach. A historical RTOG dose escalation trial settled on the maximum tolerated single-fraction doses as 24 Gray (Gy), 18 Gy and 15 Gy for lesions ≤ 20 mm, 21–30 mm and 31–40 mm in maximum diameter, respectively [15,16]. However, for larger lesions (>20 mm), 18–15 Gy or less could be detrimental with regard to local control (LC), and a higher dose results in an increased risk of radionecrosis (RN) [17].

**Figure 2:**
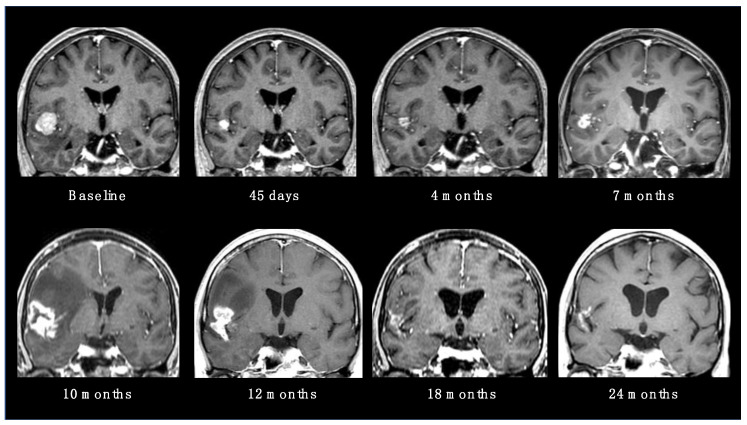
Radionecrotic evolution of a BM after SRS during a 24-month neuroradiological follow-up period.

RN is considered the main late complication of SRS, occurring from within a few months to years after radiation (up to 80% of cases have been reported within 3 years) [18]. The incidence rate is variable in the literature, usually ranging from 5 to 25% [19]. Possible risk factors are the volume of healthy brain tissue exposed to high doses of radiation, previous brain RT, concomitant ST and some specific histologies (such as ALK-translocated lung adenocarcinoma) [20]. The typical neuroradiological finding, a contrast-enhanced alteration with intralesional necrosis and perilesional edema, is not easily distinguishable from local tumor recurrence (Figure 2). Usually, gyriform lesions and edema with marginal or solid enhancement are more suggestive of tumor recurrence or a viable tumor. However, these patterns still have a low specificity and sensitivity [19].

For this reason, modern magnetic resonance (MR) sequences and functional imaging are essential for the differential diagnosis. In particular, a relative cerebral blood volume (rCBV) < 0.71 is highly suggestive of radionecrosis. Diffuse-weighted imaging is also highly efficient and even superior to rCBV in distinguishing recurrent tumors from RN. Moreover, positron emission tomography (PET) imaging with amino acid tracers may be extremely useful, because of the high amino acid uptake by tumor cells. Tracers, including Carbon-11 methionine, Fluoro-l-thymidine and Fluoroethyltyrosine, have been tested with promising results and very high sensitivity (up to 100%) and specificity (up to 93%) rates. [19]. Most RN presentations are asymptomatic and allow a conservative strategy, based on neuroradiological observation. When necrotic lesions cause symptoms, therapeutic options vary from corticosteroid administrations to the use of bevacizumab, up to the need for surgery in the case of large and edemigenous lesions [21].

Hypofractionated stereotactic RT (SRT) represents the ideal solution to improve LC without increasing the risk of RN related to focal RT in the management of large BMs. SRT delivers a lower dose per fraction in few (generally 3 or 5) fractions, thus allowing it to simultaneously deliver a higher biologically effective dose (BED) to the tumor and a lower radiobiological effect to the surrounding healthy brain tissue. Retrospective series of large brain lesions treated with SRT have accumulated over the years, and these have demonstrated better rates of LC and RN compared to SRS, as shown in two meta-analyses [22,23]. Ongoing randomized studies could definitively confirm these benefits (NCT03697343, NCT05222620, NCT05346367).

The international collaborative “HyTEC” (Hypofractionated Treatment Effects in the Clinic) project published data on tumor control probability (TCP, considered as the probability of LC) [24] and normal tissue complication probability (NTCP, considered as the risk of RN) [25] to guide dose and fractionation choices for SRS/SRT in BMs. The authors concluded that a 18–24 Gy single-fraction SRS is optimal for BMs ≤ 20 mm, while fractionated SRT should be preferred for lesions > 20 mm [24,25]. In clinical practice, the prescription dose for SRT treatment generally ranges from 24 to 30 Gy in three to five fractions. In addition to different RT dose fractionations, also the exposure of healthy brain tissue should be weighted in order to minimize the risk of RN. The recent UK consensus on normal tissue dose constraints suggests keeping the brain volume below 10–15 cc (including the target volume) for a maximum dose of 12 Gy (V_12Gy_) in a single fraction, whereas the dose received by 20 cc (D_20cc_) of the brain should not exceed 20 Gy and 24 Gy when SRT is delivered in three and five fractions, respectively [26]. Table 1 shows some of the most commonly adopted SRS/SRT schedules for BMs, with the relative BED and equivalent dose in 2 Gy/fraction (EQD2) for tumor and brain parenchyma, alongside the dose constraints predictive for RN.

For further optimization of the therapeutic index in the management of large BMs, a novel approach named staged SRS (SSRS) has been investigated. It consists of delivering SRS in two or three fractions separated by an interval of a few weeks. Treatment replanning between each session is necessary to adapt RT volumes to the progressive shrinkage of the BM. Thus, a higher final BED on the tumor is achievable, while simultaneously maintaining a lower risk of RN. Despite the promising results of preliminary reports [27,28,29,30,31,32,33,34,35], further evidence is awaited before there is wide adoption of SSRS in clinical practice. Most studies included patients with BMs from different primary tumors, with NSCLC representing the most frequent (30–100%) histology. On the other hand, SCLC patients deserve specific consideration (as reported in the specific section below), as they were rarely included in studies investigating the role of SRS and SRT (≈5%).

### 2.2. SRS for Limited BMs

The management of oligometastatic brain disease, considered as a stage with a limited number (maximum 3–4) of BMs, indicating an intermediate status between the absence of brain lesions and a disseminated presentation, has evolved considerably during the past few decades. Historically, WBRT was considered the mainstay for BMs regardless of the number of lesions. Starting from the 1980s, several randomized clinical trials were conducted to investigate the role of focal treatments (either surgery or SRS) in this setting, enrolling a large number of NSCLC patients [36,37,38,39,40].

Firstly, the RTOG 9508 trial randomized patients with 1–3 BMs to receive WBRT + SRS versus WBRT alone. That study showed a better LC in the SRS group (1 y LC: 81% vs. 71%, *p* = 0.01), which resulted in an improved OS for patients with a single BM (median OS: 6.5 vs. 4.9 months, *p* = 0.039) [40]. The proven effectiveness of focal RT raised the question of the feasibility of omitting WBRT after SRS for oligometastatic BMs, possibly avoiding the neurocognitive dysfunctions related to extended radiation fields.

A second generation of randomized trials and a meta-analysis of individual patient data explored this scenario [41,42,43,44,45]. The results, despite the different primary endpoints considered, were univocal, showing similar OS rates despite a lower intracranial disease control in patients receiving SRS alone. At the same time, WBRT was confirmed to negatively impact both on neurocognitive function and quality of life (QoL) [42,43,44,46]. Furthermore, three secondary analyses of these trials, conducted using NSCLC patients, failed to prove a definitive advantage of additional WBRT over SRS after stratification for DS-GPA [47,48,49].

In conclusion, data from the aforementioned studies led to the progressive omission of WBRT for oligometastatic BMs in favor of focal therapies, which demonstrated a more favorable risk–benefit ratio (durable LC, lower toxicity and identical OS). Moreover, the feasibility of repeating SRS in case of further relapses (see the section dedicated to SRS retreatment below), with limited or no toxicity, encouraged this epochal transition in the treatment paradigm for oligometastatic BMs. Nowadays, according to current international guidelines, SRS alone is the central pillar for the management of a limited number of BMs [50,51,52,53].

### 2.3. SRS for Polymetastatic BMs

**Figure 3:**
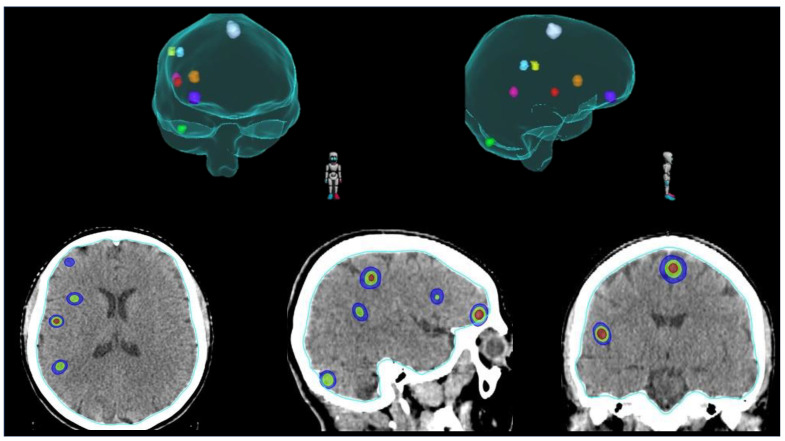
Example of SRS for the treatment of multiple (≥5) BMs.

Although WBRT is still considered the standard RT approach for patients with multiple (≥5) BMs [54], the current trend is to propose the use of SRS also in this particular setting. Exploiting technological advances, we are now able to treat all the macroscopic metastatic deposits with focal RT (Figure 3), potentially improving LC and minimizing the exposure of uninvolved brain parenchyma [55].

When SRS is offered with a LINAC-based approach, a single isocenter may be used to simultaneously treat multiple BMs, in order to reduce the delivery time and improve patients’ compliance [56,57,58,59].

Several studies investigated the role of SRS for polymetastatic BMs and provided the proof of principle for its feasibility, efficacy and safety. Yamamoto et al. led the first retrospective analyses, showing that carefully selected patients with up to 10 or more BMs are not unfavorable candidates to receive SRS alone [60,61,62,63,64]. Other multicenter retrospective [65] and prospective studies [66,67] confirmed these results, with LC, OS and toxicity rates comparable to those observed in oligometastatic patients. Despite the studies cited so far, robust data from phase III trials are still lacking to date. A Dutch trial, investigating WBRT versus SRS for patients with 4–10 BMs, prematurely interrupted the enrollment (mainly as a result of patients’ and doctors’ preference for SRS) and no definitive results were produced on the non-inferiority of SRS in terms of OS and brain-failure-free survival [68]. Another randomized controlled trial, presented at the American Society for Radiation Oncology (ASTRO) annual meeting in 2020, randomized patients with 4–15 non-melanoma BMs to receive WBRT or SRS. The radiosurgical treatment was associated with a reduced risk of neurocognitive deterioration without compromising OS (median OS: SRS 10.4 months vs. WBRT 8.4 months, *p* = 0.45) [69].

Ongoing phase III trials comparing hippocampal-avoiding WBRT (HA-WBRT) versus SRS (NCT03075072, NCT03550391, NCT04277403; see Table 2) will provide further evidence for this scenario. Meanwhile, SRS should be offered to carefully selected patients with multiple BMs, while also taking into consideration impactful variables such as BM tumor volume [70,71] and BM velocity [72,73].

### 2.4. SRS Reirradiation

The use of upfront SRS with deferral of WBRT makes close MRI follow-up essential. Serial neuroimaging allows monitoring of the outcomes of treated lesions, in terms of LC and RN, and early detection of the eventual development of new intracranial metastases [74]. Literature data estimate a risk for local relapse of approximately 10–20% and for distant brain recurrence of up to 50% after SRS alone for BMs [41,42,43,44,45]. Although there is no consensus, SRS represents a valid salvage strategy for both local and distant intracranial recurrence, presenting considerable advantages over the other treatment modalities, such as WBRT, surgery and ST.

Multiple courses of SRS to treat new BMs appeared to be safe and effective for carefully selected patients, and it allowed them to avoid or delay WBRT [75,76]. Series conducted on NSCLC patients confirmed the feasibility of this approach [77,78]. The trial NRG-BN009 is an ongoing phase III trial investigating the impact of HA-WBRT in addition to SRS on patients with BM velocity ≥ 4 new brain metastases/year at the time of a first or second distant brain relapse after upfront SRS (NCT04588246; see Table 2).

The scenario of SRS reirradiation, in the strict sense, meaning the radiosurgical retreatment of a locally recurrent BM, is more controversial. Although surgery, when feasible, is generally considered the best salvage strategy after local relapse [79], repeated SRS is a viable alternative. Retrospective series [80,81] and two meta-analyses [82,83], including patients with BMs from different primary tumors, showed encouraging data in terms of LC, although retreatment carried a significant risk of RN. A recent large retrospective analysis validated these results for an NSCLC population [84]. A dose of 16–18 Gy in single fraction emerged as the most used, with the general belief that dose escalation may lead to an improvement in LC [81]. According to a recent study, fractionated SRT seemed to be a valuable option also for reirradiation, comparing favorably to single-fraction SRS with regard to LC and RN risk (1 y local failure: 27.7%; 1 y RN: 15.6%) [85]. REMASTEr (NCT05124912; see Table 2) and Re-TREAT (NCT05126875; see Table 2) are ongoing studies whose results are awaited to better define the role of SRS reirradiation.

To conclude, SRS may represent a valid solution in the spectrum of salvage therapies after prior SRS, allowing avoidance of the issues related to surgery or WBRT in this setting.

## 3. SRS and Surgery

Surgery plays a central role in the treatment of BMs, remaining the gold standard for large and edemigenous lesions. It ensures a decompressive effect with immediate symptom relief and, when required, provides histological diagnosis and molecular determination [51,52,53]. Despite these advantages, the LC of resected BMs is not durable after surgery alone, with recurrence rates reaching 50% [86]. Furthermore, there is a risk of leptomeningeal dissemination (LMD) of tumor cells intrinsic to the surgical procedure [87]. In this scenario, RT may represent not only an alternative but also a complementary solution to surgery, in order to improve clinical outcomes. A paradigm shift has occurred also in the adjuvant setting, with the historic WBRT approach being abandoned due to the lack of survival benefits [43,86,88], in favor of postoperative focal treatments that maximize LC without an excessive neurotoxicity.

### 3.1. Adjuvant SRS

Focal RT of the surgical cavity after surgical resection of BMs represents the current standard of care in many centers and is recommended by the most recent guidelines [51,52,53]. Using one (SRS) or a few (SRT) sessions of ablative RT, it is possible to reduce the risk of local recurrence associated with exclusive surgery. In the last two decades, several retrospective series investigated the role of adjuvant SRS, and a recent meta-analysis, pooling 3458 patients from 50 studies, revealed high LC rates (1 y LC: 83.7%) and a low toxicity profile (1 y RN rate: 6.9%) [89]. Two randomized trials confirmed the efficacy and safety of this approach [90,91]. Mahajan and colleagues randomized patients to receive adjuvant SRS versus observation after gross total resection of 1–3 BMs. The results showed an excess of local recurrence in the observation group (1 y freedom from local recurrence: 43% vs. 72% in the SRS arm, *p* = 0.015) [90]. In the multicenter NCCTG N107C/CEC.3 study, adjuvant SRS was compared to adjuvant WBRT for patients with a single resected BM. WBRT resulted in better overall intracranial control at 1 year compared to the SRS group (81.5% vs. 40.7%, *p* = 0.003) [91,92]. In fact, no difference was observed in terms of LC between the two arms, according to a secondary analysis of the trial presented at the ASTRO 2022 meeting (1 y LC: 79.2% vs. 86.5%, *p* = 0.099) [93]. On the other hand, the preservation of neurocognitive functions and QoL were significantly better in the SRS group [91,92]. For completeness, the trial showed no differences with regard to OS between the study arms [90,91,92].

Based on this evidence, adjuvant SRS of the surgical cavity has progressively become the recommended treatment in patients with resected BMs, although many uncertainties still need to be resolved. First, the optimal dose and fractionation to obtain an ideal balance between LC and the risk of RN is still under investigation. Considering that surgery is generally proposed for large symptomatic lesions, it is very common to face large postoperative volumes, which may not be adequately treated with a single-fraction SRS. As described for intact BMs, fractionated SRT seems to be the ideal solution to this problem. Several series and meta-analyses have suggested an excellent benefit–risk ratio for adjuvant SRT [94,95,96,97,98], and the completion of ongoing randomized trials is awaited for stronger evidence (NCT04114981, NCT05160818; see Table 3). Other issues related to postoperative focal treatment depend on the dynamic adaptation of the surgical cavity, which complicates the identification of target volumes and the timing of adjuvant RT. Given this heterogeneous scenario, consensus contouring guidelines published in 2017 provided technical and practical recommendations with the aim of standardizing postoperative SRS for completely resected BMs [99]. Regarding the optimal timing, an interval from surgery of 4–6 weeks is suggested [100]. Finally, adjuvant SRS is thought to be inferior to WBRT in reducing the risk of LMD after surgery, with a reported incidence of up to 35% after postoperative focal RT [101]. LMD may even appear with a distinct nodular pattern, which is less likely to be symptomatic and has a better OS rate than the classical linear pattern when treated with adjuvant SRS [9].

To conclude, adjuvant SRS/SRT is considered a valuable treatment strategy after surgical resection of BMs, although further evidence from larger retrospective studies and randomized trials is eagerly awaited to clarify the role of focal treatments in this setting.

### 3.2. Neoadjuvant SRS

**Figure 4:**
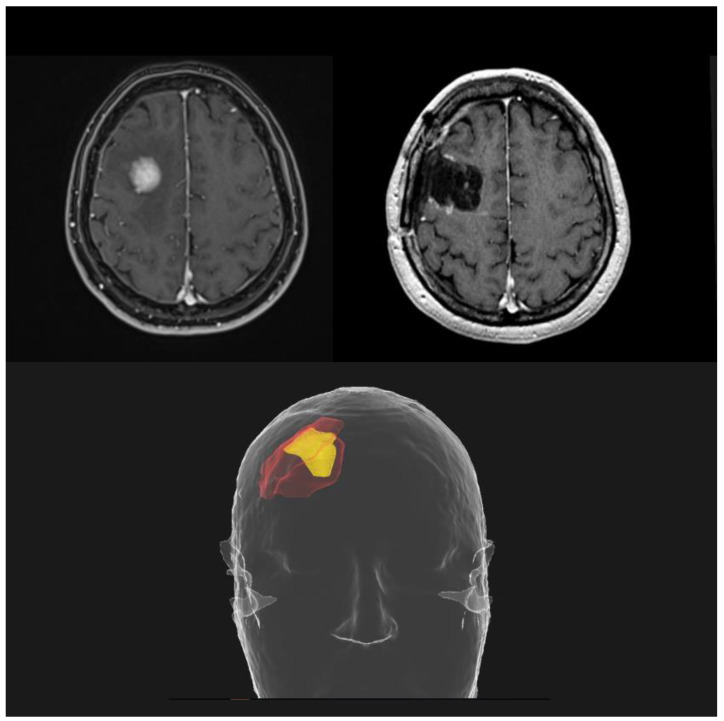
Potential reduction in treatment volumes using a preoperative SRS approach (yellow), compared to postoperative SRS (red).

Neoadjuvant SRS represents the new frontier in the management of resectable BMs. All the critical issues related to adjuvant SRS led to the investigation of a preoperative strategy; the rationale is to sterilize any microscopic disease before the macroscopic resection of the brain lesion. A better definition of the irradiation target with shrinkage of the treatment volumes (Figure 4), a reduced risk of RN and LMD and a contraction of the overall treatment time are all potential advantages over postoperative focal RT.

On the other hand, the main limitations of a neoadjuvant approach are the risk of irradiating a lesion with an unproven histological confirmation and delaying surgery in those urgent cases that require rapid intervention to control a symptomatic lesion. Neoadjuvant RT is typically performed as a single fraction of 16 Gy, given a few days before surgery; more recently, fractionated SRT (median doses of 24–30 Gy in 3–5 fractions) was proposed with the intention to improve the therapeutic index [102].

Several single-arm studies collected retrospective and prospective data of patients treated with neoadjuvant SRS and showed favorable oncological outcomes and toxicity profiles (low rates of RN and LMD) [103,104,105,106,107,108,109,110,111]. The trial PROPS-BM, a multicenter cohort study at five American institutions, represents the largest published series in this setting. With a median time between SRS and surgery of 1 day (range 1–3 days), the median prescribed dose at the 80% isodose line was 15 Gy. The treatment was effective (1 y LC: 85%; 1 y distant control: 63.7%; 1 y OS: 57.7%) with a low rate of complications (LMD rate: 7.9%; RN rate: 7.1%) [112].

Ongoing studies, particularly randomized phase III trials comparing neoadjuvant versus adjuvant SRS (NCT03741673, NCT03750227, NCT04474925, NCT05438212, NCT05545007; see Table 3), will provide further information about this promising approach.

## 4. SRS and Systemic Therapy (ST)

Historically, ST played a secondary role in the management of BMs, due to the blood–brain barrier limiting the penetration to the CNS of the traditional chemotherapeutical agents. Except for highly chemosensitive histologies, such as SCLC, intracranial response rates to chemotherapy were considered unsatisfactory [50]. The modern targeted therapies, particularly immune checkpoint inhibitors (ICIs) and tyrosine kinase inhibitors (TKIs), markedly improved the prognosis for specific oncological settings and strengthened the role of ST in the treatment of BMs.

In NSCLC, new-generation targeted agents, such as osimertinib in the case of EGFR mutation other than exon 20 insertions [113,114], and alectinib, ceritinib, brigatinib, lorlatinib and ensartinib in the case of ALK translocation [115,116,117,118,119,120], have shown excellent results for patients with BMs, with intracranial overall response rates (ORRs) ranging between 78% and 91%, as well as encouraging tolerability results. Moreover, *ROS1*, *RET* and *NTRK* rearrangements, *MET* exon 14 skipping mutation and *KRAS* G12C mutations represent further molecular markers targetable by drugs, for which novel specific inhibitors have shown encouraging intracranial activity [121,122,123,124,125,126,127,128,129]. However, all these prospective data derive from small subsets of patients enrolled in clinical trials with treated and/or asymptomatic BMs, and this limitation should be considered when translating them to clinical practice. Table 4 summarizes these findings.

At the same time, ICIs demonstrated a potential intracranial activity for NSCLC patients both when used alone (ORR range: 16.4–55.9%) [133,134,135,136,137,138] and in association with chemotherapy [139,140].

This led to the investigation of a multimodal approach, integrating SRS and modern ST to exploit both these highly effective treatment solutions in different BM scenarios. In addition to the potential synergistic effect achievable with the concurrent administration of SRS and ST [141,142], there is growing interest regarding the possibility of starting upfront ST and delaying or omitting focal treatments [143,144]. To date, no evidence from randomized trials is available to define the optimal combination in terms of timing, safety and efficacy for the new ST options and SRS, requiring a case-by-case evaluation within multidisciplinary discussions.

### 4.1. SRS and Immune Checkpoint Inhibitors (ICI)

The advent of ICIs improved the prognosis for NSCLC at different disease stages [145,146,147] and significantly reduced the incidence of BMs (incidence of BMs: 6.5% after durvalumab versus 11.8% after placebo, according to the long-term results of the PACIFIC trial) [148], thus drawing attention to the CNS activity of these drugs.

In recent years, the association of SRS and immunotherapy for the treatment of BMs was tested in several retrospective series. Three meta-analyses showed better LC and OS when the two treatment modalities are concurrently administered (1 y LC: 73.2–89.2%; 1 y OS: 64.6–68%) versus a sequential approach (1 y LC: 67.8–69.5%; 1 y OS: 42.7–58%) [149,150,151]. In addition to the increased LC, the synergy between RT and ICIs also seemed to improve distant intracranial control, reducing the risk of developing new brain lesions [152]. Moreover, the literature has reported cases of specific immune responses after SRS-ICI, such as abscopal effect, pseudoprogressions or immune-related adverse events, occasionally related to a better prognosis [153,154]. Although all these studies were predominantly conducted on melanoma patients, NSCLC series confirmed their results [155,156,157].

Regarding toxicities, some reports described an increased RN risk (reported RN rates: 16–37.5%) after concurrent SRS and ICIs [158,159,160]. Despite this, meaningful data have argued in favor of the feasibility of concurrent therapies maintaining acceptable RN rates (<10% at 1 year) [161,162].

Larger prospective trials, possibly limited to a specific histology, are needed to better characterize the efficacy and toxicity profiles of SRS-ICI strategies (NCT02696993, NCT04042220, NCT04047602, NCT04291092, NCT04427228, NCT04787185, NCT04889066, NCT05522660, NCT05703269; see Table 5). The evidence available to date is insufficient to strongly recommend the use of upfront ICIs alone, deferring RT when it would be indicated according to current practice.

### 4.2. SRS and Tirosine-Kinase Inhibitors (TKIs)

Oncogene-addicted NSCLC is characterized by a particularly high incidence of BMs (about 20% in the case of EGFR-mutated and up to 40% for ALK-rearranged tumors at the diagnosis) [163]. TKIs revolutionized the prognosis in this scenario and gained an important role in patients presenting with BMs. The excellent CNS efficacy of new-generation EGFR and ALK inhibitors allowed the consideration of modern ST as an effective single-modality treatment, reserving RT as a consolidation or salvage option in case of persistent or progressive disease [164,165,166]. In light of the aforementioned results of subgroup analyses from randomized trials [113,114,115,116,117,118,119], upfront TKIs are currently accepted by international guidelines for selected patients with multiple asymptomatic BMs from EGFR-mutated and ALK-translocated NSCLC [51,52,53].

Nevertheless, RT remains a highly effective focal approach and its omission in favor of ST alone may be detrimental, both in terms of intracranial control [167] and survival [168], even in the oncogene-addicted population. Notably, Magnuson et al. demonstrated that SRS followed by first-generation EGFR inhibitors significantly improves OS compared to EGFR inhibitors alone (median OS: 46 vs. 25 months, *p* < 0.001) in TKI-naïve patients with newly diagnosed BMs. Patient selection was extremely important, as SRS seemed to play a key role only in the oligometastatic setting (82% of the patients in the SRS + EGFR inhibitors group had less than 5 BMs) [168].

Therefore, another strand of interest aims to analyze the combination of SRS and TKIs, outlining the potential synergistic effect and the toxicity profile. The first studies investigated WBRT and old-generation TKIs, showing promising but inconclusive results [169,170,171]. The paradigm shift towards the new generation of TKIs with better CNS penetration and the progressive expansion of focal RT techniques have further modified the treatment landscape in this setting.

For EGFR-mutated patients, a few retrospective series investigated the association between osimertinib and SRS, showing a positive impact on clinical outcomes and a potential OS benefit in patients with upfront oligometastatic brain involvement [172,173,174]. In particular, a recent study by Zhao and colleagues showed that upfront cranial therapies like SRS or surgery in addition to osimertinib may provide extra survival benefits compared to osimertinib alone (median OS: 39 vs. 27 months, *p* = 0.041) [173].

ALK translocation, as well as the other molecular drivers targetable by drugs in NSCLC (ROS1, MET, BRAF, RET, HER2 and NTRK), are less common [175], making it difficult to accumulate robust data on combined SRS-TKI treatment in these specific settings [171,176]. Conversely, KRAS mutations are detected in 25–30% of patients with lung adenocarcinoma. However, the use of KRAS inhibitors has been expanding only in the last few years [128,129], and their combination with SRS represents an interesting frontier to explore in the future.

In the context of polymetastatic BMs, different strategies can be adopted for these patients. The efficacy of modern ST can be exploited with the administration of upfront TKIs in order to downstage CNS disease, avoiding upfront WBRT and converting some patients into SRS candidates [177]. On the other hand, upfront SRS remains feasible also for multiple BMs, with promising results according to a recent multicenter analysis on patients with EGFR-mutated and ALK-translocated NSCLC [178].

To conclude, SRS and TKIs are two effective treatment solutions for the management of BMs from oncogene-addicted NSCLC, allowing patients to avoid WBRT even in the case of multiple lesions. The results from ongoing studies investigating the combination of focal RT and modern target inhibitors (NCT03497767, NCT03769103, NCT04905550, NCT04908956, NCT05033691, NCT05236946, NCT05522660; see Table 6) are awaited to better define the risks and benefits of the concurrent administration.

### 4.3. SRS and SCLC

The management of BMs from SCLC deserves specific considerations. SCLC is a poor prognosis disease, with a great tendency to brain metastatic dissemination and intracranial progression [179]. In this scenario, WBRT is still considered the standard of care, delivered alternatively with prophylactic intent [180,181] or to treat macroscopic BMs, even for patients with a low CNS involvement [182]. Historically, SCLC histology was considered an exclusion criterion from randomized trials investigating the role of SRS for BMs, resulting in a lack of prospective evidence [183]. Nevertheless, the development of advanced neuroimaging, with the possibility of omitting prophylactic cranial irradiation (PCI) in favor of MRI surveillance [184], and the introduction of ICIs in combination with standard chemotherapy regimens [185,186,187,188], paved the way for a paradigm shift potentially including focal RT techniques.

In the last few years, an increasing number of institutions published retrospective studies exploring the safety and the effectiveness of SRS for SCLC patients [189,190,191,192,193,194,195,196,197]. FIRE-SCLC is a cohort study representing the largest series available to date; over 700 patients with BMs from SCLC received first-line SRS, reporting favorable outcomes and no inferiority in terms of OS after propensity-score-matched comparison with first-line WBRT. In particular, patients developing oligometastatic brain involvement seemed to have a favorable prognosis when treated with SRS (median OS: 11 months for patients receiving SRS to a single BM) [198]. Recently, a systematic review and meta-analysis was conducted with the aim of strengthening these findings. This study showed longer OS following SRS compared with WBRT ± SRS boost (HR: 0.85, 95% CI 0.75–0.97) or WBRT alone (HR: 0.77; 95% CI 0.72–0.83) [199].

Until the results from the ongoing trials (NCT03297788, NCT04804644, NCT03391362, NCT04516070; see Table 7) are available, SRS can be considered a therapeutic option for carefully selected patients with SCLC BMs.

## 5. Towards Novel Integrated Treatments: The Role of Molecular Profiling and Microenvironment Characterization

### 5.1. A—Molecular Profile of Lung Cancer BMs: Genomic Characterization

The molecular profile of lung cancer BMs has been investigated through comprehensive, multimodal “-omic” analyses to better characterize the driver alterations enabling BM establishment, progression and resistance to treatments [200]. Most studies have employed next-generation sequencing (NGS) assays to investigate genetic alterations including single-nucleotide variations (SNVs), small insertions and deletions (InDels) and gene rearrangements. Within a cohort of 552 NSCLCs, Wang et al. characterized 153 matched BM specimens, demonstrating for the first time that RET fusions increase the risk of BM development. Furthermore, the significance of EGFR exon 21 mutations and exon 19 frameshift deletions as predictive alterations of BM development was also confirmed [201,202]. The authors also assessed the incidence of ALK alterations [201], reporting the retention of ALK-EML4 translocations in BMs compared with primary tumors, and a rate of about 3–11% for ALK amplifications in these samples [203,204]. A whole exome sequencing (WES) approach on a series of 40 samples, comprising both primary lung adenocarcinoma (AC) and BMs, showed a discrepancy in the mutational landscape between primary and secondary lesions. BMs were found to be characterized by a higher level of genomic alterations both in terms of copy number alterations (CNAs) and single-nucleotide variants (SNVs), with a low rate (8.3%) of shared mutations between BM and primary lung cancer. A further recent comprehensive genomic characterization of lung AC BMs investigated new putative private driver alterations and, in accordance with other studies [205], confirmed the preservation of primary AC driver mutations involving TP53, KRAS, STK11, KEAP1 and EGFR. Moreover, a phylogenetic analysis revealed that the accumulation of somatic mutations, as well as the genetic divergence and heterogeneity between primary tumors and BMs, are the result of the concurrent evolution of primary subclones and metastatic ones which can be explained through a parallel progression model [204].

Concerning relevant CNAs, in addition to the well-established role of the loss of homozygosity of CDKN2A/B, BMs can harbor either focal amplifications of MYC (8q24.21) or YAP1, BIRC3 and TMEM123 (11q.22.2), or of a cluster of metalloproteinases including MMP13, EDNRA, ARHGAP10 and NR3C (4q31.23). Interestingly, the comparison between 58 BMs and their matched primary ACs demonstrated that the mentioned chromosomal alterations (8q24.21, 11q22.2, 4q31.23) are only present in the BMs, determining a clonal divergency during the metastatic process and potentially favoring a low response to RT, as previously reported for other tumors [206,207]. Finally, patient-derived xenograft (PDX) mouse models confirmed these results demonstrating the role of YAP1, MMP13 and MYC, whose overexpression confers a greater propensity to grow in brain microenvironments, probably due to MYC-mediated activation of stemness-like phenotypes and pathways [208,209].

Molecular characterization of a series of 79 stage IV lung squamous cell carcinomas (SQCCs) and matched BMs revealed that the latter retain the PTEN loss of function from their primitive tumors [210]. PTEN loss of function has been described as an important alteration in lung cancer BMs because it mediates the activation of anti-apoptotic pathways such as NF-kB, COX-2, MAPK and PI3K, causing a higher resistance to therapies which induce apoptosis like RT [211]. SQCC-BMs retain primitive tumors’ alterations, but also harbor novel private ones, such as the 5q31 gain where proto-cadherins reside [212].

The proto-oncogene MET is an important player involved in metastatic processes, given its key role in the epithelial–mesenchymal transition (EMT). Stella et al. investigated the mutational landscape of MET in a case series of 68 samples of lung cancer (NSCLCs and neuroendocrine tumors—NETs), showing a higher rate of mutations and amplifications in BMs, a finding associated with higher RT resistance [213]. These findings are especially relevant for NETs since fewer data are available regarding these tumors due to the low availability of specimens caused by the lower rate of surgical resections [214]. Despite these challenges, Dono et al. evaluated the genomic alterations of a series of BMs from different tumors, including 10 BMs from SCLC. Genetic analysis, also performed using data from the COSMIC and TGCA databases, demonstrated that SCLC-BMs are enriched in alterations of specific genes: the epigenetic regulator ARID1A, component of the SWI/SNF complex, and the proto-oncogene FGF10 [215]. Mouse models have also been developed to perform in vivo characterization. BM cannot be investigated in genetically engineered mouse models (GEMMs) since the timeframe for their development is not sufficient before death occurs because of primary tumor progression and/or other metastasis development. On the other hand, cell-derived xenograft (CDX) and orthotopic mice, generated by injecting patient-collected circulating tumoral cells (CTCs) [214,216], develop BMs, allowing the characterization of these entities [216,217,218]. Moreover, the development of SCLC-organoids is a novel tool which might improve the molecular characterization of this entity; Quaranta et al. developed a protocol to establish cerebral organoids of SCLC metastasis to recapitulate the dissemination process and to characterize BM profiles the interactions between SCLC cells and brain parenchyma cells, and the response to therapeutical regimens, including RT [219].

**Figure 5:**
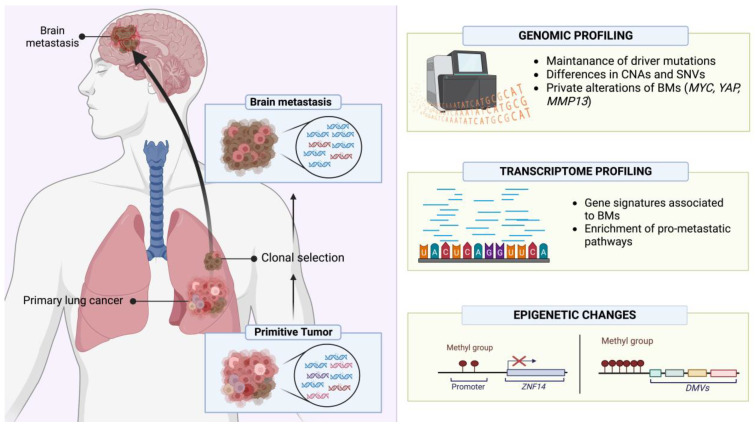
Schematic overview of the molecular characteristics of lung cancer BMs investigated through “-omic” analyses. Created with BioRender (https://www.biorender.com/, accessed on 13 September 2023).

An overview of lung cancer BMs’ molecular profile investigated through “-omic” analyses is provided in Figure 5.

### 5.2. B—Molecular Profile of Lung Cancer BMs: Transcriptomic and Epigenetic Features

Genomic characterization is just one type of tumor molecular profiling. Indeed, transcriptomic and epigenetic characterization are also critical to achieve a comprehensive molecular dissection of a tumor. Gene expression analysis demonstrated the activation of pro-invasion/metastatic signaling in BMs, especially of Rap1, whose role during invasion processes has been previously reported for primary brain tumors. Moreover, BMs showed an enrichment in pathways involved in RT resistance, such as PI3K/AKT [220,221,222,223]. Kramer et al. profiled 91 fresh-frozen samples from patients affected by NSCLC, among whom 32 had developed BMs, to assess the risk of patients to develop BMs and to find new tumoral biomarkers. The RNA-seq analysis demonstrated a signature of 22 transcripts which was highly associated with BM risk. Moreover, the pathway analysis found an enrichment of genes related to oxidative phosphorylation, in particular those associated with the respiratory chain complex I [224]. To elucidate the risk of BM development, Koh et al. analyzed 36 lung AC cases with BMs and 36 lung AC cases without BM, comparing the expression of a large series of immune-related genes. The analysis found a gene signature of 11 immune-related genes (DPP4, ICAM1, RARRES3, CD74, CSF2, HLA-DMB, ICAM5, MUC1, CCL18, RORC and ICAM4) associated with a higher risk of BM development, and this signature was further validated by immunohistochemical stainings to verify protein expression [225]. A further study, focused on BM cells retrieved from the cerebrospinal fluid (CSF) of lung adenocarcinoma patients, studied the gene signatures associated with BM development. This transcriptome analysis showed that CTCs in CSF harbor an enrichment in genes associated with cell adhesion, especially those necessary for the interaction with vessel cells and tissue colonization, as well as EMT, extracellular matrix remodeling and metabolic activities, highlighting how these pathways are essential for both the tumor spread and seeding in distant sites [226] (Figure 5).

DNA methylation profiling is now an important, clinically relevant tool, allowing the determination of tumor types according to their epigenetic signatures. Epigenetic analysis could also allow the identification of specific BM hallmarks. Therefore, Karlow et al. analyzed the methylation profile of BMs, comparing matched ‘trios’ of specimens retrieved from a series of 12 patients with lung adenocarcinoma and including lung normal tissue, lung primary cancer tissue and BMs. The profiling identified differentially methylated regions in metastatic lesions showing a gain of DNA methylation at specific sites. In particular, the promoter of ZNF154, a zinc finger related to the suppression of the metastatic process, was found to be hypermethylated, and thus silenced, in BM specimens. The analysis of DNA methylation valleys (DMVs), which are a hotspot of regulatory regions for several developmental genes [227], demonstrated that BMs are characterized by a gain of methylation, with the enrichment of heterochromatin markers such as H3K4me1, H3K9me3, H3K4me3, H3K27ac and H3K27me3, as well as a decrease in H3K36me3 methylation score, determining a suppression of polycomb repressor complex and boosting the metastatic process [228] (Figure 5).

These studies highlight that BMs are molecularly distinct entities compared to their primary tumors and harbor specific molecular traits associated with resistance to treatments, including RT. Further knowledge regarding these mechanisms will hopefully allow the current clinical gap to be filled and disclose novel effective targets for both BM prevention and treatment.

### 5.3. What’s Next? The Importance of Tumor Microenvironment and the Interplay with RT

Metastasis development is a strongly selective multistep process in which the role of the tumor microenvironment (TME) is crucial. Whether in primary or secondary sites, the interactions between tumoral cells and resident host cells shape the development and progression of neoplasms, also modulating their tropism to specific distant organs [229]. Regarding lung cancer, CNS is the predominant metastatic site and BMs occur frequently [230]. Cancer cells manage to cross the blood–brain barrier, hijacking its protective mechanisms and invading brain parenchyma to establish initial micrometastatic seeding [231]. Multiple cellular types within the TME are reprogrammed to promote cancer progression; astrocytes undergo several alterations in cellular morphology and gene expression through reactive gliosis, promoting the proliferation and chemoresistance of BMs [232,233]. Tumor-associated macrophages (TAMs) are driven towards M2 subtype differentiation, sustaining a more aggressive tumoral phenotype through the production of several cytokines capable of inducing angiogenesis, extracellular matrix remodeling and immunosuppression [234]. Dendritic cells (DCs) assume an immunosuppressive role in NSCLC, enhancing the ability of Treg cells to suppress T lymphocyte proliferation [235]. Even tumor-associated neutrophils (TANs) can differentiate in the pro-tumoral phenotype N2, which contributes to ECM remodeling, immune evasion, proliferation and angiogenesis [236].

In this setting, RT can induce changes in the TME that may modulate both tumor response and resistance through a subtle, highly complex balance between the activation and the suppression of the immune system [237]. Irradiation exerts an immunostimulating activity by increasing NK cell cytotoxicity, tumor infiltration by CD8+ cytotoxic T lymphocytes and M1 differentiation of macrophages, while reducing the level of infiltrating regulatory T cell (Treg) lymphocytes; these changes ultimately result in an inhibition of tumor growth [238]. In addition, irradiation increases the amount of major histocompatibility complex (MHC) molecules on the surface of the cells, and the secretion of several factors necessary for the activation of dendritic cells that initiate innate and adaptive immune responses. Unfortunately, pro-neoplastic changes are also induced, including the release of molecules, like the anti-inflammatory cytokine TGF-β, which promote tumor growth and invasion, inhibit cytotoxic cell functions and alter the autophagic and necrotic signaling, increasing the energetic supply of BM cells and suppressing death-inducing mechanisms [239,240]. On the other hand, the induced necrosis and necroptosis may activate the immune system against neoplastic cells through the establishment of a pro-inflammatory and pro-apoptotic microenvironment caused by TNF-α, INF-γ and IL-2 released by CD8+ T cells and NK cells [241,242]. These conflicting and intertwined relationships show the complex nature of the effects exerted by RT and highlight the therapeutic potential of targeting these interactions.

The main promoters of immunosuppression within the TME are Tregs, since they are resistant to RT and driven to proliferate by PD-1 [243]. Tregs are responsible for the production of TGF-β that suppresses the cytotoxic activity of CD8+ T cells, promotes the conversion of CD4+ T cells in Tregs and drives the differentiation of cancer-associated fibroblasts (CAFs), which contribute to the anti-apoptotic signaling by stimulating the PI3K pathway [244,245]. To counteract this mechanism, a high-dose regimen in hypo-fractionated stereotactic radiation therapy (HSRT) has been proposed to increase the number of CD8+ T cells, attenuate the release of TGF-β by CD4+ T cells and also reduce the number of Tregs [246].

Macrophages, especially the M2 subtype activated by inflammatory cytokines, are also involved in the establishment of an immunosuppressive TME. Even though the RT effect on macrophage differentiation needs to be fully elucidated, it has been reported that irradiation with either single or fractionated doses can activate both M1 and M2 macrophages, ultimately leading to tumor growth [247]. However, the RT-induced changes are still controversial, since an in vitro and in vivo study reported that irradiation of macrophages within the TME with 10 Gy, despite leading to an increase in the number of M1 cells and a reduction in M2 cells, nevertheless caused the upregulation of pro-survival pathways, such as NFκB and Bcl-2, increasing the invasive and metastatic phenotype [248]. Conversely, exposure to a low dose of radiation may cause reprograming of macrophages into M1 cells, which triggers infiltration of cytotoxic T lymphocytes (CTLs) into the TME [249].

Exposure of the TME to radiation causes secretion of IFN-γ, which induces the expression of MHC I and VCAM-1, resulting in lymphocyte trafficking and increasing anti-tumor immunity [250,251]. CTLs represent the most important anticancer cells in the TME, and highly radioresistant tumors may prevent the infiltration of lymphocytes [252]. Their activity directly depends on antigen presentation by DCs, whose antitumoral function can be enhanced by exposure of tumors to radiation and the subsequent upregulation of Toll-like receptors (TLRs) [253]. The higher presentation of damage-associated molecular patterns (DAMPs) to CTLs increases the release of anti-cancer cytokines, facilitating cytotoxicity against cancer cells [254]. Also, in this case, the high radiation doses administered in HSRT (at 10 Gy/fraction) may further amplify the release of immune-stimulating antigens and antigen presentation by DCs [255].

Given this evidence, several experimental studies have been conducted to modulate the TME and sensitize cancer cells to chemotherapy and/or RT [256]. The first pathway to be targeted was DNA repair, using inhibitors such as olaparib and veliparib, which are currently employed in clinical practice.

Since inflammation is a further important mechanism involved in tumor progression and resistance, strategies focused on its modulation have also been actively investigated. For instance, the enzyme COX-2 is known to be produced at higher levels in the TME and plays a key role in tumor growth and metastatization, so its targeting could be feasible for sensitizing cancer cells to RT [257]. It was demonstrated that inhibition of COX-2 with celecoxib increases apoptosis following irradiation in some types of cancer cells, and clinical studies confirmed the role of COX-2 targeting in tumor resistance, showing promising results in terms of relapse-free survival and OS in patients with breast and lung cancers, although these data were not confirmed in other clinical trials [258,259,260,261].

Hypoxia is another important player in radioresistance and tumor progression. It has been suggested that HIF-1 is involved in both radioprotection and radiosensitization since it can activate p53 and apoptosis in irradiated cells, but also stimulates VEGF secretion to promote tumor vascularization [262]. Since the radioprotective effect of HIF-1 is highly dependent on p53 activity, in some conditions like the mutation and inactivation of p53, HIF-1 inhibition may be a promising strategy for the sensitization of tumors to RT [262,263]. However, the radioresistance induced by hypoxia could represent an issue in cases of treatment with anti-angiogenetic therapies against VEGF and its associated receptors, which may induce hypoxia. Actually, the concomitant irradiation of lung xenografts with the VEGF-receptor inhibitor cediranib induced tumor apoptosis and necrosis, as well as higher levels of hypoxia, but this was not associated with an acquired radioresistance since the maintenance of the inhibitor after RT treatment prevented the contribution of hypoxic cells to tumor regrowth and enhanced radiation response [264].

Considering the significant impact that the TME exerts on cancer progression, it is clear that novel strategies are warranted to modulate these interactions and counteract the pro-oncogenic mechanisms. As mentioned above, Tregs are more radioresistant than CTLs, and an increase in the Tregs to CTLs ratio is responsible for the establishment of an immunosuppressive environment in the TME. The main mechanism by which Tregs inhibit CTLs is the PD-1/PD-L1 axis, which is frequently reprogrammed by tumors as an escape mechanism from the immune system [265]. Thus, targeting this pathway in combination with RT may boost immune detection by cytotoxic cells and the exhaustion of Tregs within the TME, providing a biological rationale for the clinical efficacy of these therapeutic combinations [266,267]. Nowadays, several tumor types are being treated with immune checkpoint inhibitors (ICIs), and indeed immunotherapy with pembrolizumab and nivolumab has become a standard treatment option for many malignancies, including metastatic NSCLC [268,269]. Unfortunately, the BM response to anti-PD-L1 antibodies is poorer compared to that of primary lung lesions, probably due to a markedly decreased amount of PD-L1+ tumor infiltrating lymphocytes (TILs) in secondary lesions [270]. Moreover, exposure of cancer cells to radiation may cause upregulation of PD-L1, leading to resistance to subsequent doses of RT. Also, the genomic subset of NSCLC more prone to develop BMs could be associated with a diminished response to immunotherapy [271]; it has been reported that patients with EGFR mutations or ALK/ROS1 fusions present a characteristic low tumor mutational burden (TMB) and, despite the observed high expression of PD-L1 in tumoral cells, these patients present minimal benefits from ICIs and are associated with a short progression-free survival [272]. On the contrary, KRAS-mutated NSCLC patients present a high TMB and an increased infiltration of PD-L1-positive lymphocytes, resulting in an inflammatory TME which enables a better response to immunotherapy [273]. Multiple targeting of immune response modulators can also be envisaged; for instance, it was demonstrated that the simultaneous inhibition of PD-L1 and T cell immunoglobulin mucin-3 (TIM-3) in combination with RT may be more effective for tumor control [266].

Concerning other CNS cell types, Monteiro et al. demonstrated that BMs are promoted by high levels of S100A9 expressed by cancer cells following the inflammatory response caused by the interaction with reactive astrocytes. This molecule mediates resistance to RT through the NF-κB pathway, and its signaling could be impaired by the inhibition of its receptor RAGE [274]. S100A9 could thus be a relevant target for improving RT efficacy and also be exploited as a predictive biomarker since circulating levels of this protein before and immediately after WBRT treatment correlate with survival. Supporting this potential role, its inhibitor FPS-ZM1 showed a radiosensitizer effect independently of primary tumor origin and without side effects on healthy brain tissue, representing an innovative therapeutic option.

In conclusion, understanding the role exerted by the BM TME is critical to devise new strategies to lift the tumor-induced immunosuppression, improving the efficacy of both RT and ST, and ultimately extending disease control and survival in this challenging clinical setting.

**Figure 6:**
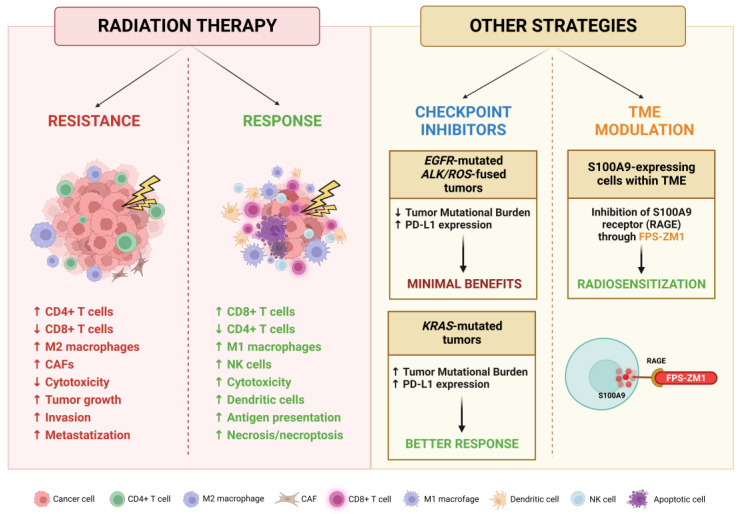
Schematic representation of effects exerted by RT on BM TME and potential strategies for its targeting. Created with BioRender.

An overview of RT influence on BM TME and the proposed modulation strategies is provided in Figure 6.

## 6. Conclusions

SRS/SRT represents a cornerstone of lung cancer BM treatment, enabling LC in a high percentage of patients with lower rates of side effects compared to older modalities. SRS/SRT methods are also undergoing a fast-paced technical evolution, expanding their role in novel settings like patients with multiple BMs. The landscape of ST has also been completely reshaped in the last decade thanks to the deployment of ICIs and novel targeted therapies for oncogene-addicted tumors. Nevertheless, multiple open issues remain to be addressed, such as how to integrate these therapeutic tools most effectively for each patient. Finally, the revolution brought by ICIs has shown that neoplastic cells are just one side of the coin. Current translational research is highlighting the importance of both the BM-specific molecular profile of BMs and of TME interactions. These data will hopefully result in the identification of new vulnerabilities and potential targets, to improve RT efficacy and enable novel therapeutic strategies.

## Figures and Tables

**Table 1:** Most commonly adopted SRS/SRT schedules and relative BED for tumor (α/β 10 Gy) and brain parenchyma (α/β 2 Gy), with corresponding brain dose/volume constraints for RN.

Dose/Fractions	BED Gy_10_	EQD2 Gy_10_	BED Gy_2_	EQD2 Gy_2_	Constraints
SRS (single fraction)					V_12Gy_ < 10–15 cc
21 Gy/1 fx	65.1	54.25	241.5	120.75	
18 Gy/1 fx	50.4	42	180	90	
SRT (three fractions)					D_20cc_ < 20 Gy
27 Gy/3 fx	51.3	42.75	148.5	74.25	
24 Gy/3 fx	43.2	36	120	60	
SRT (five fractions)					D_20cc_ < 24 Gy
30 Gy/5 fx	48	40	120	60	
25 Gy/5 fx	37.5	31.25	87.5	43.75	

Abbreviations: BED, biologically effective dose; EQD2, equivalent dose in 2 Gy fractions; SRS, stereotactic radiosurgery; fx, fractions; SRT, stereotactic radiotherapy.

**Table 2:** Ongoing randomized clinical trials investigating the role of SRS for intact BMs in different settings.

NCT Identifier	Study Phase	Patients Estimated	Population	Experimental Arm	Standard Arm	Primary Endpoint
**SRS vs. SRT**						
NCT03697343 (FSRT)	III	382	Large BMs (2–4 cm)	SRT (48 Gy/12 fx)	SRS (15–18 Gy/1 fx)	Time to local progression
NCT05222620 (FRACTIONATE)	II	69	Large BMs (2–4 cm)	SRT	SRS	LC; RN
NCT05346367 (SAFESTEREO)	II	130	BMs	SRT (30–35 Gy/5 fx)	SRS (15–24 Gy/1 fx)	LC; RN
**SRS > 4 lesions**						
NCT02953717 (CAR-Study B)	NA	81 (actual enrollment)	11–20 BMs	GKRS (18–25 Gy/1 fx)	WBRT (20 Gy/5 fx)	Cognitive decline at 3 mo.
NCT03075072 (BWH/DFCI, Boston)	III	196	5–20 BMs	SRS/SRT (1–5 fx)	WBRT (30 Gy/10 fx)	QoL
NCT03550391 (CCTG CE.7)	III	206	5–15 BMs	SRS (18–22 Gy/1 fx)	HA-WBRT (30 Gy/10 fx)	OS; neurocognitive PFS
NCT04277403 (HipSter)	III	150	4–15 BMs	HA-WBRT-SIB (30 Gy/12 fx—SIB 51 Gy)	SRS/SRT (18–22 Gy/1 fx—30 Gy/5 fx)	iPFS
NCT04891471 (WHOBI-STER)	NA	100	≥5 BMs	SRS (15–24 Gy/1 fx)/SRT	WBRT (30 Gy/10 fx)	Neurocognitive-QoL changes
NCT05378633 (CyberChallenge)	II	190	4–15 BMs	SRS	WBRT	OS; QoL
**SRS reirradiation**						
NCT04588246 (NRG-BN009)	III	350	post-SRS DIR (BMV ≥ 4 BMs/y)	SRS + HA-WBRT	SRS	Time to neurologic death
NCT05124912 (RE-MASTEr)	NA	154	A: post-SRS LR B: post-SRS RN	A: re-SRT + LITT B: LITT + steroids	A: LITT B: steroids	A: Time to intracranial progression/death B: time to steroid cessation
NCT05126875 (Re-TREAT)	II	44	Post-SRS LR	Re-SRS		LC

Abbreviations: NCT, national clinical trial; SRS, stereotactic radiosurgery; SRT, stereotactic radiotherapy; BMs, brain metastases; fx, fractions; LC, local control; RN, radionecrosis; NA, not applicable; GKRS, GammaKnife radiosurgery; WBRT, whole brain radiotherapy; QoL, quality of life; HA, hippocampal avoidance; OS, overall survival; PFS, progression-free survival; SIB, simultaneous integrated boost; iPFS, intracranial PFS; DIR, distant intracranial recurrence; BMV, brain metastases velocity; LR, local recurrence; LITT, laser interstitial thermal therapy.

**Table 3:** Ongoing randomized clinical trials investigating the role of SRS complementary to surgery.

NCT Identifier	Study Phase	Patients Estimated	Population	Experimental Arm	Standard Arm	Primary Endpoint
**Adjuvant SRS**						
NCT04114981 (Alliance A071801)	III	242 (actual enrollment)	Resected BMs	SRT (3–5 fx)	SRS (1 fx)	Local recurrence free survival
NCT05160818 (SATURNUS)	III	126	Resected BMs	SRT (30–35 Gy/5 fx)	SRS 12–20 Gy/1 fx)	1 y LC
**Neoadjuvant SRS**						
NCT03741673 (MDACC)	III	110	Resectable BMs	Neoadjuvant SRS	Adjuvant SRS	1 y LMD free rate
NCT03750227 (Mayo Clinic)	III	140	Resectable BMs	Neoadjuvant SRS	Adjuvant SRS	CNS composite endpoint event
NCT04422639 (University of Arkansas)	II	104	Resectable BMs	Neoadjuvant SRS/SRT	Adjuvant SRS/SRT	Time to CNS composite event (LC, symptomatic RN, LMD)
NCT04474925 (AHS Cancer Control Alberta)	III	88	Resectable BMs	Neoadjuvant SRS	Adjuvant SRS	1 y LC
NCT05438212 (NRG-BN012)	III	236	Resectable BMs	Neoadjuvant SRS	Adjuvant SRS	Time to composite adverse endpoint (LR, LMD, RN)
NCT05545007 (SUPPORT)	III	146	Resectable BMs (>2 cm)	Neoadjuvant SRT (27 Gy/3 fx)	Adjuvant SRT (27 Gy/3 fx)	1 y LMD rate

Abbreviations: NCT, national clinical trial; SRS, stereotactic radiosurgery; BMs, brain metastases; SRT, stereotactic radiotherapy; fx, fractions; LC, local control; LMD, leptomeningeal disease; CNS, central nervous system; RN, radionecrosis; LR, local recurrence.

**Table 4:** Intracranial activity of selected new-generation tyrosine kinase inhibitors (TKIs) in TKI-naïve advanced NSCLC patients.

Study	Target and Molecule	Untreated Brain Mts Patients	icORR Measurable Lesions (Total Number of Patients)	icORR Measurable Plus Non Measurable (Total Number of Patients)	mDOR Measurable Lesions	mDOR Measurable Plus Non Measurable
FLAURA (Soria JC, 2018 [130])	*EGFR* mutation—Osimertinib	Yes, neurologically stable	76% (53)	-	13.8 (95% CI, 10.8–20.2)	-
ALEX (Gadgeel S, 2018 [115])	*ALK* rearrangement—Alectinib	Yes, asymptomatic	85.7% (7) prior RT 78.6% (14) no prior RT	36% (25) prior RT 74.4% (39) no prior RT	-	NR (95% CI, 14.8-NR) prior RT; NR (95% CI, 13.4-NR) no prior RT
ALESIA (Zhou C, 2019 [131])	*ALK* rearrangement—Alectinib	Yes, asymptomatic (leptomeningeal disease included)	94% (17)	73% (44)	NE	NE
ALTA-1L (Camidge RD, 2018 [118])	*ALK* rearrangement—Brigatinib	Yes, neurologically stable	78% (18)	66% (47)	27.9 (95% CI, 5.7–NE)	27.1 (95% CI, 16.9–42.8)
CROWN (Solomon BJ, 2022 [119])	*ALK* rearrangement—Lorlatinib	Yes, asymptomatic	83% (18)	65% (37)	NR (95% CI NR–NR)	NR (95% CI NR–NR)
eXalt3 (Horn L, 2021 [120])	*ALK* rearrangement—Ensartinib	Yes, asymptomatic	64% (11)	NA	NA	NA
ALKA-372-001, STARTRK-1, STARTRK-2 (Drilon A, 2023 [122])	*ROS1* rearrangement—Entrectinib	Yes, asymptomatic or neurologically stable	80% (25)	52.1% (48)	12.9 (95% CI, 6.8–22.1)	12.9 (95% CI 7.1–22.1)
LIBRETTO-001 (Drilon A, 2023 [122])	*RET* rearrangement—Selpercatinib	Yes, if asymptomatic or neurologically stable	85% (26)	NA	9.4 (95% CI, 7.4–15.3)	NA
ARROW (Griesinger F, 2022 [123])	*RET* rearrangement—Pralsetinib	Yes, if asymptomatic or neurologically stable	70% (10)	NA	10.5 (95% CI, 5.5–12.6)	NA
ALKA-372-001, STARTRK-1, STARTRK-2 (Doebele RC, 2020 [126])	*NTRK1,2,3* rearrangement- Entrectinib	Yes, asymptomatic or neurologically stable	55% (11) *	NA	NE (95% CI, 5.0-NE)	NA
Pooled analysis of phase 1–2 trials (Hong DS, 2020 [127])	*NTRK1,2,3* rearrangement- Larotrectinib	Yes, asymptomatic	66% (3) *	75% (12) *	NA	NA
KRYSTAL-1 (Negrao MV, 2023 [132])	*KRAS* p.G12C mutation—Adagrasib	Yes, asymptomatic or neurologically stable	NR	42.1% (19)	NR	12.7 (95% CI, 3.9-NE)
VISION (Mazieres, J, 2023 [125])	*MET* exon 14 skpping mutation—Tepotinib	Yes, asymptomatic or neurologically stable	57% (7)	60% (15)	NR	NR
GEOMETRY mono-1 (Wolf J, 2020 [124])	*MET* exon 14 skipping mutation—Capmatinib	Yes, neurologically stable	NR	54% (13)	NR	NR

* Patients with NTRK-rearrangement positive solid tumors, including NSCLC. Abbreviations: icORR, intracranial objective response rate; RECIST, response evaluation criteria in solid tumors; mDOR, median duration of response; CI, confidence interval; RT, radiation therapy; NR, not reached; NE, not estimated; NA, not assessed.

**Table 5:** Ongoing prospective studies investigating the combination of SRS and ICIs for NSCLC patients.

NCT Identifier	Study Phase	Patients Estimated	Population	Experimental Arm	Comparator	Primary Endpoint
NCT02696993 (MDACC)	I-II (non-random)	88	BMs from NSCLC	A: nivolumab + SRS B: nivolumab + WBRT C: nivolumab + ipilimumab + SRS D: nivolumab + ipilimumab + WBRT		MTD (phase I); 4 mo iPFS (phase II)
NCT04042220 (Medical University of Vienna)	NA (cohort)	200	BMs from NSCLC or melanoma	A: GKRS + ICI B: GKRS + ICI + glucocorticoids C: GKRS alone		OS
NCT04047602 (RADREMI)	NA (single arm)	42	BMs from NSCLC (1–10)	ICI + reduced dose SRS		Symptomatic RN rate
NCT04291092 (Zhejiang Cancer Hospital)	II (single arm)	63	BMs from NSCLC	camrelizumab + CT + cranial RT (SRS/WBRT)		6 mo PFS
NCT04427228 (MIGRAINE)	II (random)	74	BMs (1–10) from solid tumors,	ICI + SRT (27 Gy/3 fx)	ICI + SRS (18–20 Gy/1 fx)	RN rate
NCT04787185 (STRAIT-LUC)	NA (cohort)	50	BMs from NSCLC (1–10)	ICI + SRS/SRT		≥G3 toxicity
NCT04889066 (University of Texas Southwestern Medical Center)	II (random)	46	BMs from NSCLC (1–10)	durvalumab + SRT (24–27 Gy/3 fx)	durvalumab + PULSAR (personalized ultra-fractionated stereotactic adaptive RT	intracranial clinical benefit (CR, PR, SD)
NCT05522660 (USZ-STRIKE)	III (random)	190	Asymptomatic BMs from melanoma or NSCLC;	ST (ICI or TKI) + SRS	ST (ICI or TKI) alone	iPFS
NCT05703269 (HYPOGRYPHE)	NA (random)	244	BMs from NSCLC, melanoma, breast or RCC	ICI + SRT (3–5 fx)	ICI + SRS (1 fx)	≥G2 ARE (adverse radiation effects)

Abbreviations: NCT, national clinical trial; BMs, brain metastases; NSCLC, non-small-cell lung cancer; SRS, stereotactic radiosurgery; WBRT, whole brain radiotherapy; MTD, maximum tolerated dose; iPFS, intracranial progression-free survival; NA, not applicable; GKRS, GammaKnife radiosurgery; ICI, immune checkpoint inhibitors; OS, overall survival; RN, radionecrosis; CT, chemotherapy; SRT, stereotactic radiotherapy; LC, local control; LR, local relapse; CR, complete response; PR, partial response; SD, stable disease; TKI, tyrosine-kinase inhibitors; RCC, renal cell carcinoma.

**Table 6:** Ongoing prospective studies investigating the combination of SRS and TKIs for NSCLC patients.

NCT Identifier	Study Phase	Patients Estimated	Population	Experimental Arm	Comparator Arm	Primary Endpoint
NCT03497767 (OUTRUN)	II (random)	80	BMs from EGFR-mutated NSCLC	osimertinib + SRS (at least 1 lesion)	osimertinib alone	1 y iPFS
NCT03769103 (LUOSICNS)	II (random)	76	BMs from EGFR-mutated NSCLC	osimertinib + SRS	osimertinib alone	iPFS
NCT04905550 (RadiAI-CNS)	II (single arm)	50	BMs from EGFR-mutated NSCLC	almonertinib + cranial RT (SRS/WBRT)		iPFS
NCT04908956 (STEREO)	II (single arm)	60	Oligomts EGFR-mutated NSCLC	osimertinib + SRS/SBRT (primary + all mts)		≥G2 toxicity, PFS
NCT05033691 (LUNG-OSIME-SRS)	NA (random)	162	Asymptomatic BMs from EGFR-mutated NSCLC	osimertinib + SRS	osimertinib alone	iPFS
NCT05236946 (Tata Mermorial Hospital)	III (random)	190	Asymptomatic BMs from oncogene-mutated NSCLC	TKI + cranial RT (SRS/WBRT)	TKI alone	2 y iPFS
NCT05522660 (USZ-STRIKE)	III (random)	190	Asymptomatic BMs from melanoma or NSCLC	ST (ICI or TKI) + SRS	ST (ICI or TKI) alone	iPFS

Abbreviations: NCT, national clinical trial; BMs, brain metastases; NSCLC, non-small-cell lung cancer; SRS, stereotactic radiosurgery; iPFS, intracranial progression-free survival; WBRT, whole brain radiotherapy; oligomts, oligometastatic; SBRT, stereotactic body radiotherapy; mts, metastases; TKI, tyrosine-kinase inhibitors; ICI, immune checkpoint inhibitors.

**Table 7:** Ongoing prospective studies investigating the role of SRS in SCLC with BMs.

NCT Identifier	Study Phase	Patients Estimated	Population	Experimental Arm	Standard Arm	Primary Endpoint
NCT03297788 (ENCEPHALON)	II	56	1–10 SCLC BMs	SRS/SRT (18–20 Gy/1 fx–30 Gy/5 fx)	WBRT (30 Gy/10 fx)	Neurocognition (HVLT-R test)
NCT03391362 (Dana-Farber Cancer Institute)	II	100	1–10 SCLC BMs	SRS/SRT (18–20 Gy/1 fx–30 Gy/5 fx)		Neurological death
NCT04516070 (MDACC)	II	70	1–5 SCLC BMs	SRS		3 mo cognitive decline rate
NCT04804644 (NRG-CC009)	III	200	1–10 SCLC BMs	SRS/SRT	HA-WBRT	Time to neurocognitive failure

Abbreviations: NCT, national clinical trial; SCLC, small-cell lung cancer; BMs, brain metastases; SRS, stereotactic radiosurgery; SRT, stereotactic radiotherapy; fx, fractions; WBRT, whole brain radiotherapy; HVLT-R, Hopkins Verbal Learning Test-Revised; HA, hippocampal avoidance.
